# Supplementary material for: NF-κB oscillations translate into functionally related patterns of gene expression
Source: eLife. 2016 Jan 14;5:e09100. doi: 10.7554/eLife.09100 (PMC4798970; doi:10.7554/eLife.09100)
Supplement: Supplementary file 1. — Listed are the primers used in Q-PCR reaction to test gene expression as reported in Figure 5 and in Figure 5—figure supplement 2 and Figure 7—figure supplement 1. DOI: http://dx.doi.org/10.7554/eLife.09100.048 [file elife-09100-supp1.doc]

| Sequences of primers used in RT-PCR quantification of nascent and mature transcripts (murine) | | |  |
| --- | --- | --- | --- |
| Gene name | Sequence (5’ -> 3’) | Annealing temperature (°C) | |
| Ikba fwd | CTTGGCTGTGATCACCAACCAG | 65 | |
| Ikba rev | CGAAACCAGGTCAGGATTCTGC |  | |
| Ccl5/Rantes fwd | ACCATATGGCTCGGACACCACT | 65 | |
| Ccl5/Rantes rev | ACCCACTTCTTCTCTGGGTTGG |  | |
| Nascent Ikba fwd | CTTGGCTGTGATCACCAACCAG | 65 | |
| Nascent Ikba rev | GTTTAAGACTCAAGTGGCCCCATC |  | |
| Nascent Ccl5/Rantes fwd | ATGGCTCGGACACCACTCCCTGCT | 66 | |
| Nascent Ccl5/Rantes rev | ACTGCTGCTCTAGGGCTCCCTGTG |  | |
| Rplp1 fwd | TCACTTCATCCGGCGACTAG | 60 | |
| Rplp1 rev | AGACCGAAGCCCATGTCATC |  | |
| Actb fwd | TGACGGGGTCACCCACACTGTGCCCATCTAACT | 60-65 | |
| Actb rev | CTAGAAGCATTGCGGTGGACGATGGAGGGACT |  | |
|  |  |  | |
|  |  |  | |
|  |  |  | |
