# Supplementary material for: NF-κB oscillations translate into functionally related patterns of gene expression
Source: eLife. 2016 Jan 14;5:e09100. doi: 10.7554/eLife.09100 (PMC4798970; doi:10.7554/eLife.09100)
Supplement: Supplementary file 2. — In this file we indicate the biochemical rates and the constants considered, which are described in the Materials and methods. We also provide the value of these rates and the bibliographic reference from which they were taken, if any. Otherwise, we motivate our selection of the value or we state that they were manually fitted. We also indicate the related model parameters resulting from the adimensionalization of the equations of the dynamics and the uncertainty degree, a measure of how uncertain each parameter value is. This is also a measure of how much we allow each parameter to vary in our exploration of the dynamics of the system and in our fittings. Notice that higher uncertainty degrees are assigned to parameters that were manually fitted or to those that are involved in reactions from our models that summarize many different biochemical processes. DOI: http://dx.doi.org/10.7554/eLife.09100.049 [file elife-09100-supp2.doc]

| Biochemical rates, constants used to build the model | | | | |  |
| --- | --- | --- | --- | --- | --- |
| **Name** | **Value** | **Reference(s) followed** | **Resulting model parameter(s)** | **Uncertainty degree *D*** | |
| K_R,I_ | 0.2 copies^-1^ s ^-1^ | Tay et al., (2010) | - |  | |
| d_R,I_ | 7.5·10^-4^ s ^-1^ | Tay et al., (2010) | d_R,I_ | 0.1 | |
| K_I_ | 0.25 copies^-1^ s ^-1^ | Approx from Tay et al., (2010) | k_I_=K_I_·K_R,I_·G_0_/(d_R,I_·NF-κB_0_) | 0.3 | |
| d_I_ | 6.7·10^-5^ s ^-1^ | Approx from Tay et al., (2010) | d_I_ | 0.2 | |
| K_on,I_ | 6.9·10^-8^ copies^-1^ s ^-1^ | Zambrano et al., (2014b) | k_on,I_=K_on,I_· NF-κB_0_ | 0.5 | |
| K_off,I_ | 1.4·10^-8^ copies^-1^ s ^-1^ | Zambrano et al., (2014b) | k_off,I_=K_off,I_· NF-κB_0_ | 0.5 | |
| S_tot_ / S_nuc_ | 3 | Our measurements | - | 0.1 | |
| A | 1.4·10^-6^ copies^-2^ s ^-1^ | Zambrano et al., (2014) | a=A·NF-κB_0_ | 0.5 | |
| d | 8.4·10^-4^ s ^-1^ | Zambrano et al., (2014) | d | 1 | |
| γ | adimensional | Tay et al., (2010) | γ | 0.2 | |
| κ | adimensional | Zambrano et al. (2014b) | κ | 0.5 | |
| P | 2.5·10^-8^ copies^-1^ s ^-1^ | Zambrano et al., (2014) | p=P·IKK_0_ | 1 | |
| d_K_ | 2.1·10^-4^ s ^-1^ | Manual fit | d_K_ | 1 | |
| NF-κB_0_ | 3·10^4^ copies | Zambrano et al., (2014) | - |  | |
| IKK_0_ | 10^5^ copies | Approx. from Tay et al., (2010) | - |  | |
| G_0_ | 2 copies | 2 copies per gene are assumed. | - |  | |
| A20_0_ | 3·10^4^ copies | Manual fit | - |  | |
| K_R,A_ | 0.2 copies^-1^ s ^-1^ | Tay et al., (2010) | - |  | |
| d_R,A_ | 7.5·10^-4^ s ^-1^ | Tay et al., (2010) | d_R,A_ | 1 | |
| K_A_ | 0.25 copies^-1^ s ^-1^ | Equal to K_I_ as Tay et al., (2010) | - |  | |
| d_A_ | 6.7·10^-5^ s ^-1^ | Manual fit | d_A_ | 1 | |
| K_on,A20_ | 6.9·10^-8^ copies^-1^ s ^-1^ | Equal to our K_on,I_ as in Tay et al., (2010) | k_on,A_ =K_on,A_· NF-κB_0_ | 1 | |
| K_off,A20_ | 1.4·10^-8^ copies^-1^ s ^-1^ | Equal to our K_off,I_ as in Tay et al., (2010) | k_off,A_=K_off,A_· NF-κB_0_ | 1 | |
| k_on0,I_ | 2.7·10^-4^ s ^-1^ | Manual fit | k_on0,I_ | 1 | |
| k_off0,I_ | 5.7·10^-4^ s ^-1^ | Manual fit | k_off0,I_ | 1 | |
| k_on0,A_ | 2.7·10^-4^ s ^-1^ | Manual fit | k_on0,A_ | 1 | |
| k_off0,A_ | 5.7·10^-4^ s ^-1^ | Manual fit | k_off0,A_ | 1 | |
| n | 1 | Manual fit | n | 1 | |
| K_S_ | 5.56 copies s ^-1^ (ng/ml of TNF-α) ^-1^ | Manual fit | Gives S(t)=2 h^-1^ for 10ng/ml | 1 | |
